# Modification of Electrospun Regenerate Cellulose Nanofiber Membrane via Atom Transfer Radical Polymerization (ATRP) Approach as Advanced Carrier for Laccase Immobilization

**DOI:** 10.3390/polym13020182

**Published:** 2021-01-06

**Authors:** Shuo Zeng, Jinwei Shi, Anchao Feng, Zhao Wang

**Affiliations:** 1 State Key Laboratory of Organic—Inorganic Composites, Beijing University of Chemical Technology, 15 North Third Ring Road, Beijing 100029, China; zengshuo715@163.com (S.Z.); shijin_wei@126.com (J.S.); 2 Beijing Laboratory of Biomedical Materials, Beijing University of Chemical Technology, 15 North Third Ring Road, Beijing 100029, China

**Keywords:** electrospinning, atom transfer radical polymerization, nanofiber membrane, enzyme immobilization

## Abstract

This study aimed to modify an electrospun regenerated cellulose (RC) nanofiber membrane by surface grafting 2-(dimethylamino) ethyl methacrylate (DMAEMA) as a monomer via atom transfer radical polymerization (ATRP), as well as investigate the effects of ATRP conditions (i.e., initiation and polymerization) on enzyme immobilization. Various characterizations including XPS, FTIR spectra, and SEM images of nanofiber membranes before and after monomer grafting verified that poly (DMAEMA) chains/brushes were successfully grafted onto the RC nanofiber membrane. The effect of different ATRP conditions on laccase immobilization was investigated, and the results indicated that the optimal initiation and monomer grafting times were 1 and 2 h, respectively. The highest immobilization amount was obtained from the RC-Br-1h-poly (DMAEMA)-2h membrane (95.04 ± 4.35 mg), which increased by approximately 3.3 times compared to the initial RC membrane (28.57 ± 3.95 mg). All the results suggested that the optimization of initiation and polymerization conditions is a key factor that affects the enzyme immobilization amount, and the surface modification of the RC membrane by ATRP is a promising approach to develop an advanced enzyme carrier with a high enzyme loading capacity.

## 1. Introduction

As important biological catalysts, enzymes have attracted growing interest for various applications including the food industry [1,2], biosensors [3,4], and wastewater treatment [5,6,7] because of their high specificity and reaction rates under mild conditions. However, a free enzyme has been proven to be difficult to recover and reuse from a reaction system, which limits its practical application for catalysis [8,9].

Previous research endeavors have revealed that the immobilization of an enzyme onto carriers by physical or chemical methods can achieve the recycling of the enzyme [10,11,12]. There are many methods for enzyme immobilization such as physical adsorption [13], covalent binding [14], and embedding [15]. The physical adsorption method has been widely used for enzyme immobilization because it does not change the enzyme’s conformation, which can retain enzyme activity. For example, Pang et al. reported that laccase was immobilized on carbon nanomaterials by physical adsorption, and it did not significantly change the enzyme’s active site conformation [16]. Another study showed that α-amylase and urease were immobilized in halloysite nanotubes by physical adsorption. The activity of the immobilized enzymes was 55% after seven cycles and retained 90% after being stored for 15 days [12]. Another study showed that when horseradish peroxidase was immobilized on amine-functionalized reduced graphene oxide nanosheets by physical adsorption to remove high concentration phenol biodegradation in wastewater, the thermal stability of immobilized horseradish peroxidase was observed and the activity retained about 75% at 50 °C after being incubated for 120 min [13]. 

In addition to the immobilization method, the carrier material also plays an important role in enzyme immobilization technology. The physical and chemical properties of the carrier material affect the interaction and binding position between the enzyme and the carrier, thereby affecting the performance of the immobilized enzyme [14]. Several carriers including diatomite [15], silica [17], chitosan [18], phospholipids [19], and electrospun fiber membranes (e.g., cellulose and its derivative membranes [20], as well as polylactic acid and polyvinyl alcohol fibers [21]) have been reported. Among them, the electrospun fiber membranes are considered as the most promising carriers for enzyme immobilization because of their high porosity and surface area, interconnectivity, and low mass transfer resistance [6,22,23,24]. 

Laccase (LAC; Enzyme Commission (EC) 1.10.3.2) is a type of oxidase that is easy obtain and has excellent properties [25]. The oxidation process is a mild one that catalyzes the one-electron oxidation of a range of inorganic and aromatic substances, such as phenols and aromatic or aliphatic amines, and turns them into the corresponding reactive radicals [26]. Therefore, laccase has attracted growing interest for various applications including food processing [27], wastewater treatment [28,29], and biodegradation [30]. Different types of electrospun fiber membranes, such as an electrospun nylon fiber mat (NFM) [26], electrospun poly (acrylonitrile-co-styrene/pyrrole) nanofibers, [31], polyacrylonitrile/montmorillonite (PAN/O-MMT) nanofibers [32], and polyurethane/regenerated cellulose- poly (2-hydroxyethyl methacrylate) (PU/RC -poly (HEMA)) nanofiber membranes [33], have been used as supports for laccase immobilization. However, most of these fiber membranes are produced from synthetic polymers, which are difficult to be degraded, thus causing severe pollution issues for the natural environment and potential health concerns for human beings. Cellulose is nature-derived biomass material with the advantages of good biocompatibility, abundance, degradable, non-toxicity, being harmless, etc. It has been widely used in oil–water separation [34], tissue engineering [35], and electrical devices [36]. However, cellulose has poor solubility in most organic solvents and cannot be directly used for electrospinning. To address this issue, cellulose acetate was used as raw material for electrospinning and then for the preparation of an RC membrane through hydrolysis and deacylation [37]. An electrospun cellulose membrane is a good candidate as a carrier material for enzyme immobilization due to its biodegradability, biocompatibility, large surface area, and low cost; however, it usually has weak interactions with most enzymes due to the smooth surface of electrospun nanofibers. Therefore, the surface modification of an electrospun nanofiber membrane is a promising approach to develop a high performance carrier for enzyme immobilization. For example, Gustav [8] reported that grafting a “3D-like” brush improved amount and activity compared to immobilization on self-assembled monolayers. They also found that the binding between the enzyme and the grafted brush was considerable when the pH was between the isoelectric points (pI) of the enzyme and the pKa of the polymer. 

Atom transfer radical polymerization (ATRP) can graft polymer brushes with a controllable molecular weight length/density on the surface of the substrate, which is an effective surface modification method [38]. The ATRP method is particularly suitable for grafting different polymer chains onto the surface of supports/carriers that contain functional groups such as hydroxyl and amino groups, including cellulose and its derivatives [20], graphene [39], poly (vinylidene fluoride) (PVDF) microporous membranes [40], and polyethersulfone membranes [41]. Notably, the surface of RC is rich in hydroxyl groups, and it can easily react with the initiator of the ATRP reaction to form a polymer chain/brush on the substrate surface.

The objective of this study was to modify an electrospun RC membrane via the ATRP method and investigate the effects of ATRP conditions (i.e., initiation and polymerization) on enzyme immobilization. The prepared surface-modified electrospun nanofiber membranes with polymer chains/brushes were employed to explore the relationship between structure and enzyme immobilization efficiency. DMAEMA (2-dimethylaminoethyl methacrylate) was selected as the monomer to be surface-grafted onto the RC nanofibers due to its effectiveness on enzyme immobilization [8,20]. Laccase was selected as the model enzyme for immobilization because it can oxidize phenol derivatives and other compounds containing aromatic moieties and has a great significance to bioremediation. The effects of monomer grafting density and amount on enzyme immobilization were explored. The results proved that a surface-modified electrospun RC membrane could be used as an excellent carrier for enzyme immobilization.

## 2. Materials and Methods 

### 2.1. Materials

Laccase (EC 1.10.3.2; from *Trametes versicolor* powder) was purchased from Sigma-Aldrich (St. Louis, MO, USA), and cellulose acetate (CA; Mn~30,000 g/mol, 39.8 wt.% acetyl content), polyacrylonitrile (PAN; Mw~150,000 g/mol), cuprous bromide (CuBr), 2-bromoisobutyryl bromide (2-BIBB), pyridine, 1,1,4,7,10,10-hexamethyltriethylenetetramine (HMTETA), and DMAEMA were purchased from Sigma-Aldrich. NaOH, CH_3_COONa, CH_3_COOH, Coomassie Brilliant Blue (G250), chloroform (CHCl_3_), and N, N-dimethylformamide (DMF) were obtained from Aladdin (Shanghai, China).

### 2.2. Preparation of Electrospun RC Nanofiber Membrane

Prior to electrospinning, the spin dopes of CA and PAN were prepared separately. The CA solution (6 wt.%) was obtained by adding CA to the mixture solvent of CHCl_3_/DMF (1/1, wt./wt.) containing 0.1 wt.% diethylamino ethyl chloride followed by stirring for 24 h at room temperature. The PAN solution (10 wt.%) was prepared by dissolving PAN in DMF followed by stirring at 60 °C for 24 h. During the electrospinning process, syringes loaded with spin dopes of CA or PAN were placed on opposite sides of the roller. Specifically, two syringes filled with the CA solution were applied with a positive voltage of 12 kV, and the flow rate was set at 1.2 mL/L. One syringe filled with the PAN solution was applied with a positive voltage of 8 kV, and the flow rate was set at 0.4 mL/L. The reason for adding electrospun PAN to the CA nanofibers was to improve the mechanical property of the whole CA membrane. Based on the feed rates and concentrations of the PAN and CA solutions, the obtained nanofiber membrane consisted of ~75 wt.% CA nanofibers and ~25 wt.% PAN nanofibers, with a uniform thickness of ~200 µm. Subsequently, the collected electrospun nanofiber membrane was immersed in an H_2_O/ethanol mixture (80/20, wt./wt.) containing 0.05 M NaOH for 24 h in order to convert CA into RC [34].

### 2.3. Initiation of RC Nanofiber Membrane

The obtained electrospun RC nanofiber membrane was cut into small pieces with dimensions of 2 by 2 cm. The membranes were first soaked in 20 mL of hexane for 20 min, and then they were transferred into a reaction solution containing 60 μL of pyridine and 100 mL of hexane at 0 °C for 15 min. Subsequently, 300 μL of 2-BIBB were dissolved in 10 mL of hexane before being gradually added into the reaction solution with a feeding rate of 20 mL/h. The final solution was then placed at 25 °C for 1, 3, and 6 h to allow for the initiation reaction. Thereafter, the membrane after initiation was thoroughly rinsed with hexane, ethanol, and deionized water. The initiated RC membranes with the reaction times of 1, 3, and 6 h were denoted as RC-Br-1h, RC-Br-3h, and RC-Br-6h, respectively. 

### 2.4. Surface-Grafting of Poly (DMAEMA) via ATRP

After initiation, the obtained membranes (i.e., RC-Br-1h, RC-Br-3h, and RC-Br-6h) were surface-grafted with poly (DMAEMA). A predetermined polymerization time was adopted to control the length/thickness of polymer chains. Firstly, 25 mL of methanol, 25 mL of deionized water, 110 μL of HMTETA, and 1.4 mL of DMAEMA were thoroughly degassed to remove oxygen through three freeze–pump–thaw cycles; 32 mg of CuBr were then added into the mixture solution under a nitrogen atmosphere. Subsequently, the mixture solution was degassed again, magnetically stirred for 1 h, and sonicated for 15 min until all of the CuBr was dissolved. Finally, the initiated RC-Br membrane was added into the above-mentioned solution, and the mixture was deoxygenated two times and then sealed with nitrogen at 30 °C for the polymerization reaction with different reaction times (i.e., 0.5, 1, 1.5, 2, and 2.5 h). The resulting poly (DMAEMA)-modified RC nanofibrous membrane was rinsed with deionized water and ethanol followed by being dried in air.

### 2.5. Characterization of Different Membranes

XPS was employed to characterize the chemical compositions, and the XPS measurements were performed with a Thermo Fisher Scientific USA instrument (Waltham, MA, USA) using a monochromatized Al Kr X-ray source (150W). The FTIR spectra of different nanofiber membranes were obtained from a Tensor 27 Fourier transform infrared spectrophotometer equipped with a Smart Orbit diamond attenuated total reflection accessory. The sample was scanned 32 times in the wavenumber range from 400 to 4000 cm^−1^. Additionally, SEM (FC-SM10, Hitachi S-4800, Ibaraki, Japan) was employed to characterize the morphological structures of the acquired nanofiber membranes.

### 2.6. Immobilization of Laccase on Nanofiber Membranes

The laccase solution was prepared by dissolving 1 g of laccase in 100 mL of a 0.05 M acetate buffer solution (pH = 5.0). Five milligrams of RC or RC-poly (DMAEMA) nanofiber membranes were placed into a 10 mL LAC solution (pH = 5.0) at 25 °C for 8 h under a shaking condition. Thereafter, the membrane was removed from the solution and rinsed with an acetate buffer solution until no laccase was detected. The concentration of laccase immobilized on RC or RC-poly (DMAEMA) was spectrophotometrically estimated with the Bradford method [42] and calculated by the following equation.

(1) $$Q_{e}=\frac{\left(C_{0}-C_{e}\right)\times V_{0}-C_{r}\times V_{r}}{M_{d}}$$

where Q_e_ is the amount of LAC on unit mass of nanofibers (mg/g); C_0_ and C_e_ are the initial and equilibrium LAC concentrations in the solution (mg/mL), respectively; V_0_ is the volume of the LAC solution; C_r_ is the LAC concentration in the buffer solution used for washing the immobilized enzyme nanofiber; V_r_ is the volume of the buffer solution; and M_d_ is the mass of the nanofiber membrane.

## 3. Results

### 3.1. Surface Modification of RC Nanofiber Membrane via the ATRP Reaction

**Figure 1:**
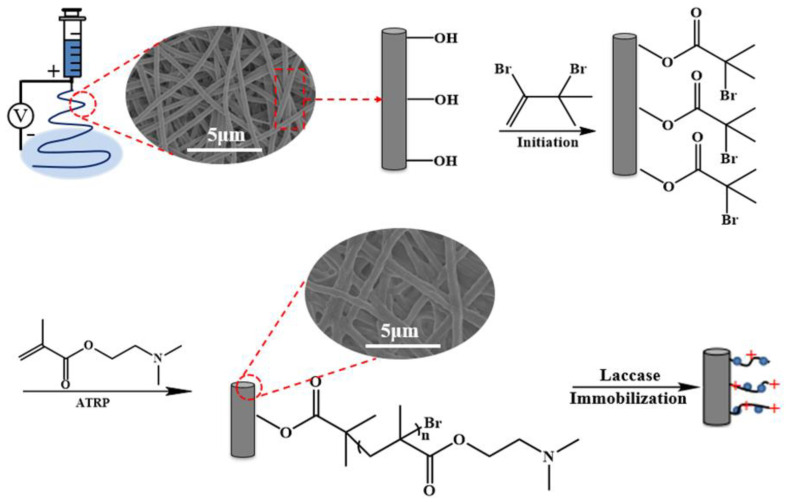
Schematic showing the electrospinning, the initiation of the electrospun regenerated cellulose (RC) nanofiber membrane, and the subsequent surface-grafting with poly 2-(dimethylamino) ethyl methacrylate (DMAEMA) chains via atom transfer radical polymerization (ATRP), as well as the immobilization of laccase on RC-poly (DMAEMA).

Figure 1 summarizes the detailed procedures for the preparation of the electrospun RC membrane, the surface modification of RC with poly (DMAEMA) by ATRP, and the immobilization of laccase.

#### 3.1.1. Initiation of RC Nanofiber Membrane

**Figure 2:**
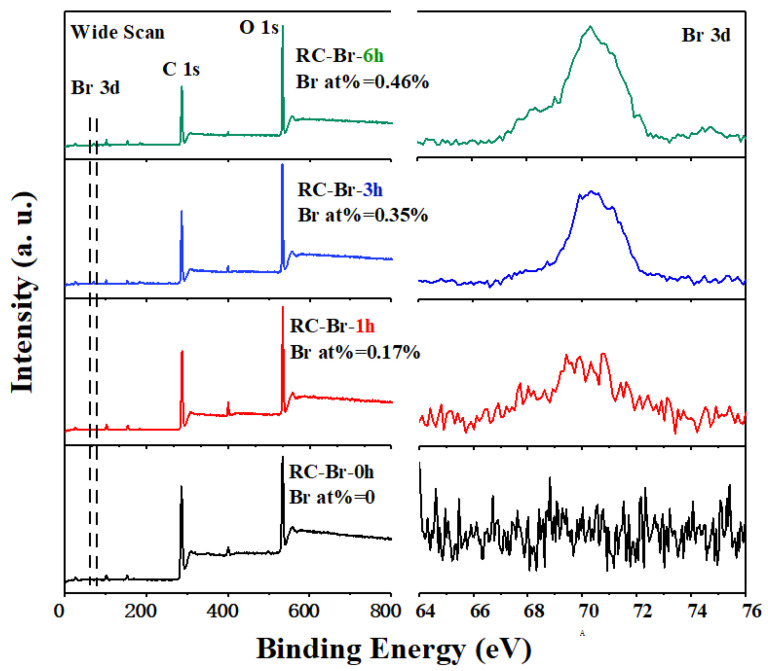
XPS spectrum of the 2-bromoisobutyryl bromide (2-BIBB)-initiated RC membranes with different reaction times of 1, 3, and 6 h.

Initiation is an important process for the ATRP reaction that provides binding points for the following polymer grafting [37]. Therefore, it determines the grafting density and polymer brush length, which affect the enzyme immobilization. To determine the effects of initiator grafting density on the RC membrane, three types of RC nanofiber membranes were prepared with the initiation reaction times of 1, 3, and 6 h. XPS and FTIR spectroscopy were employed to study the correlation of reaction time with initiation degree. Figure 2 exhibits the XPS of the initiation with 2-BIBB. The survey spectrum verified that the initiated membranes consisted of carbon (C), oxygen (O), and bromine (Br) elements, while the neat membrane only had C and O. The Br 3d spectra indicated the Br atom percentage was increased from 0.17 atom% to 0.46 atom% with the increase of the reaction time from 1 to 6 h.

**Figure 3:**
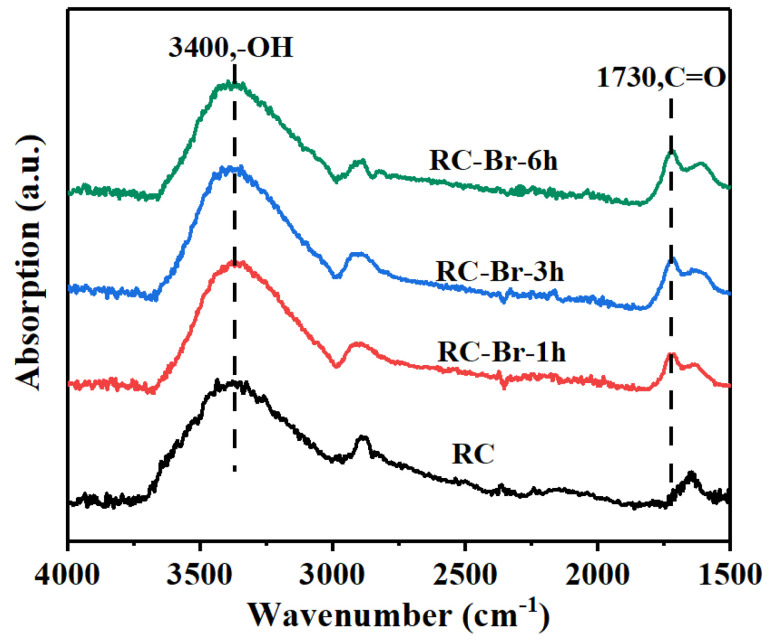
FTIR spectra of RC membranes and 2-BIBB-initiated RC membranes with different reaction times.

As shown in the FT-IR spectra (Figure 3), the RC nanofiber membrane had a characteristic band centered at the wavenumber of 3400 cm^−1^, which was attributed to the stretching vibration of hydroxyl groups. After 2-BIBB reacted with the hydroxyl groups on the RC nanofibers, the intensities of the C=O stretching vibration bands centered at 1730 cm^−1^ increased significantly, indicating the bonding between Br and tertiary carbon atoms. The results from XPS and FTIR spectroscopy confirmed the successful initiation of 2-BIBB on the RC nanofiber surface and that prolonging the reaction time improved initiation degree. All the RC membranes initiated with 2-BIBB (i.e., the RC-Br-1h, RC-Br-3h, and RC-Br-6h) proceeded with polymer grafting via the ATRP reaction.

#### 3.1.2. Surface-Grafting of Poly (DMAEMA) via ATRP

**Figure 4:**
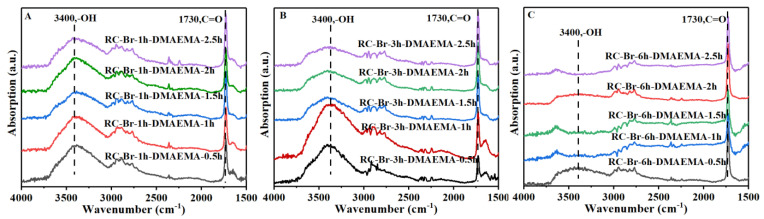
FTIR spectra of (**A**) RC-Br-1h (**B**) RC-Br-3h, and (**C**) RC-Br-6h modified by DMAEMA with different reaction times.

The 2-BIBB-initiated RC membranes (i.e., the RC-Br-1h, RC-Br-3h, and RC-Br-6h) were then surface-grafted by DMAEMA to form poly (DMAEMA) brushes by ATRP under a series of reaction times (i.e., 0.5, 1, 1.5, 2, and 2.5 h). The FTIR spectra of the different resulting poly (DMAEMA)-grafted membranes are presented in Figure 4. The bands centered at 3400, 2937, and 1730 cm^−1^ were attributed to the O-H stretching vibration, the C-H stretching vibration (in the methylene group), and the C=O stretching vibration (in the ester group), respectively. The intensities of the O-H stretching vibration bands centered at 3400 cm^−1^ were decreased, and the C=O stretching vibration bands centered at 1730 cm^−1^ were increased, prolonging both the initiation degree and the ATRP reaction time.

### 3.2. Morphological Characterization of Poly (DMAEMA)-Grafted RC Membranes

**Figure 5:**
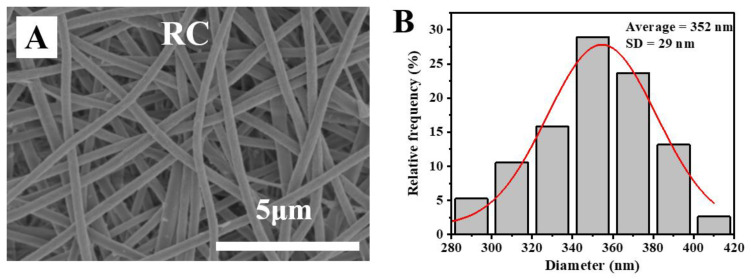
(**A**) SEM image of the electrospun RC nanofiber membrane and (**B**) its size distribution.

**Figure 6:**
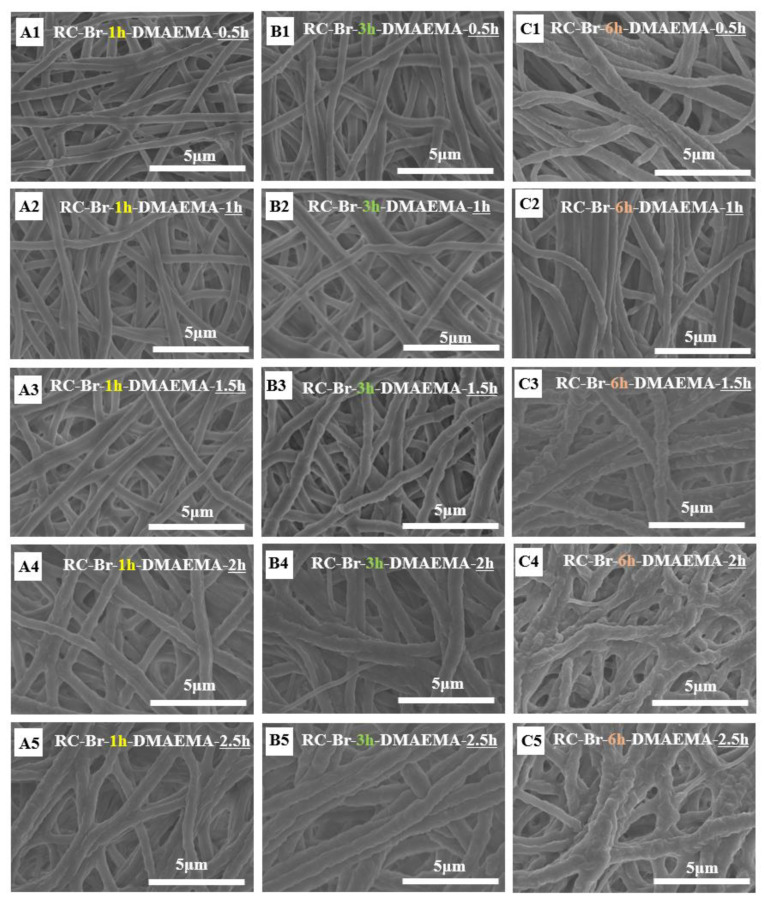
SEM images showing the morphological structures of the DMAEMA-modified RC membranes with initiation times of (**A1**–**A5**) 1 h, (**B1**–**B5**) 3 h, and (**C1**–**C5**) 6 h, as well as with different polymerization reaction times of 0.5, 1, 1.5, 2, and 2.5 h.

**Figure 7:**
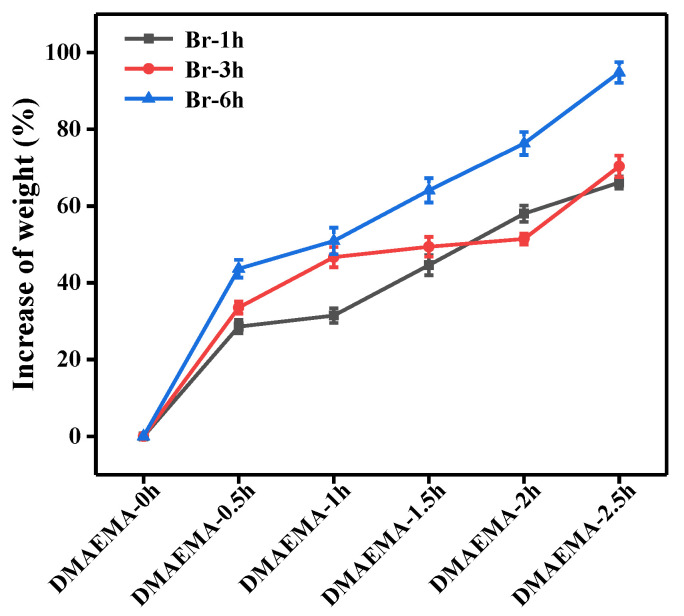
The weight change of the DMAEMA-grafted RC membranes with different initiation and grafting times.

An SEM image of an electrospun RC nanofiber and its size distribution is shown in Figure 5. The electrospun RC nanofibers were bead-free and showed a uniform and smooth surface with an average diameter of 352 nm. After being grafted with DMAEMA via the ATRP reaction, the poly (DMAEMA) macromolecular chains were evidently coated on the nanofiber surfaces, thus making the fibers thicker, as shown in Figure 6. At the same initiation degree (i.e., A1–A5), the diameter of the RC-Br-1h-poly (DMAEMA) membrane was increased when the reaction was further prolonged. The pore size was decreased with the increasing of initiation time from 1 to 3h at the same polymer grafting time of 0.5 h (i.e., A1, B1, and C1). Compared to the RC nanofibers, both the weight (Figure 7) and diameters of RC-Br-1h-poly (DMAEMA), RC-Br-3h-poly (DMAEMA), and RC-Br-6h-poly (DMAEMA) nanofibers evidently increased due to the surface-grafted polymer chains/brushes. Additionally, with the increase of fiber diameter, the pore size of the nanofibrous membrane was decreased. It has been reported that pore size among nanofibers plays an important role in enzyme attachment and transportation [6].

### 3.3. The Effects of ATRP Reaction Conditions on Enzyme Immobilization 

Laccase was selected as the model enzyme to study the effects of different ATRP reaction conditions on enzyme immobilization.

#### 3.3.1. The Effects of Initiation Degree on Laccase Immobilization

**Figure 8:**
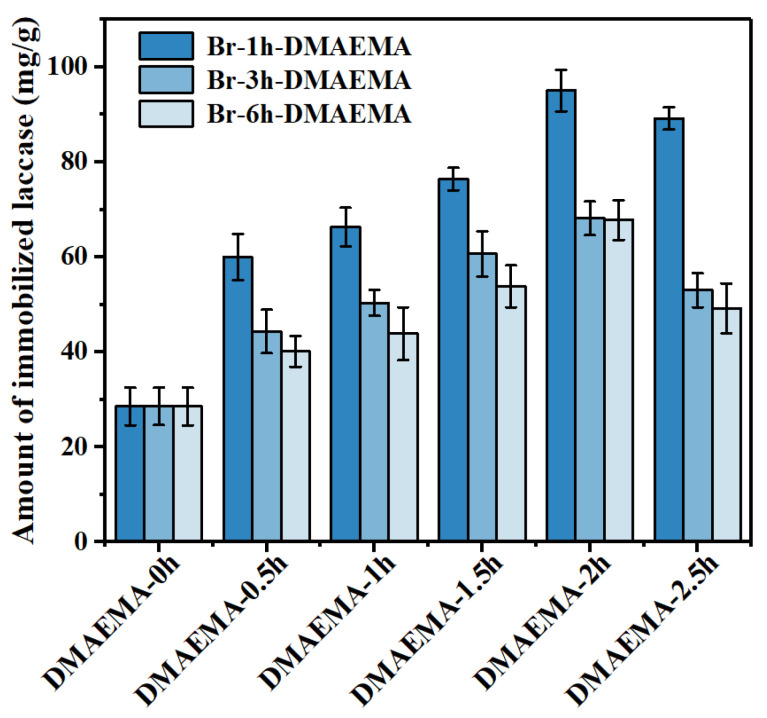
Amount of immobilized laccase for RC-Br-DMAEMA with different initiation and reaction times via ATRP.

With the increase of initiation degree, the amount of immobilized laccase was decreased, as shown in Figure 8. For example, the laccase immobilization amount on the RC membranes initiated at 1, 3, and 6 h and reacted at the same polymer grafting time of 0.5 h was decreased from about 60 to 44 and 40 mg/g, respectively. The same trend was found at different grafting reaction times (i.e., 1, 1.5, 2, and 2.5 h). A possible reason is that initiation could generate an active reaction point on the fiber surface. With longer initiation times, more active points could be generated and used for the following polymer grafting reaction. Therefore, the initiation degree could control the density of polymer grafting on the initiated membranes. If the density of polymer brushes grafted on the fiber surface is too high, there will be less space among polymer brushes, resulting in a lower laccase immobilization amount.

#### 3.3.2. The Effects of Polymer Grafting Amount on Laccase Immobilization

The influence of immobilized laccase on the different poly (DMAEMA) grafting amounts is shown in Figure 8. It is clear that the amount of immobilized laccase on the RC membrane initiated for 1 h (Br-1h) was increased with the increase of the ATRP reaction time from 0.5 to 2 h; after the laccase amount reached a maximum value, it started to decrease when the reaction time was further prolonged to 2.5 h. Noticeably, the same trends were found from those RC membranes initiated with 3 and 6 h. A possible reason is that at the same initiation degree (i.e., the same polymer brush density), increasing the polymer grafting time could have generated polymer brushes and provided more immobilization points and space (i.e., porous structure) for laccase; however, further improving the polymerization time could have continued to increase the polymer brush length, which might have become entangled at certain length and started to decrease the space among brushes. As a result, the fiber diameter became thicker and the pore size became smaller, resulting in a larger resistance for mass transfer and a lower immobilization amount. It was also shown that the amount of immobilized laccase was decreased for the RC membrane-grafted DMAEMA at 0.5 h when the initiation time was increased from 1 to 6 h. The same trends were found from the RC-Br membrane-grafted DMAEMA when the initiation time was increased from 1 and 2.5 h. It could be speculated that at the same grafting time (i.e., the same polymer brush length), increasing the initiation time could generate more grafting points, thus decreasing the pore size among fibers for laccase immobilization. 

These results indicated that both the polymer density on the fiber and the polymer length play important roles in enzyme immobilization. It is worth noting that the highest laccase immobilization amount (i.e., 95.04 ± 4.35 mg) was found from the RC-Br-1h-poly (DMAEMA)-2h membrane, indicating the optimal initiation reaction time of 1 h and polymer grafting time of 2 h. Moreover, all the RC membranes with different ATRP reaction conditions showed improved laccase immobilization amounts compared to the initial RC membrane without any treatment, thus indicating the effectiveness of ATRP treatment for the RC nanofibrous membrane on enzyme immobilization. The highest enzyme immobilization amount was approximately 3.3 times higher than the initial RC membrane. As shown in Table 1, the laccase immobilization amount of the RC-poly (DMAEMA) nanofiber membrane was superior to most of the reported carriers developed by different strategies. All the results suggested that the optimization of initiation and polymerization conditions is the key factor that affects the enzyme immobilization amount, and surface modification of the RC membrane by the ATRP reaction is a promising approach to develop advanced enzyme carriers with high enzyme loading amounts.

## 4. Conclusions

In summary, an electrospun RC nanofibrous membrane was surface-grafted with poly (DMAEMA) via ATRP with the aim to immobilize laccase on polymer brushes. Different degrees of initiation and monomer grafting amounts were prepared to study their effects on the laccase immobilization amount. The results indicated that the optimal initiation and monomer grafting times were 1 and 2 h, respectively. The highest immobilization amounts were obtained from the RC-Br-1h-poly (DMAEMA)-2h membrane (95.04 ± 4.35 mg), which increased by approximately 3.3 times compared to the initial RC membrane. The results from this work demonstrated that the optimization of the initiation and polymerization conditions is the key factor that affects enzyme immobilization amount, and an electrospun RC nanofibrous membrane surface-grafted with polymer chains/brushes via the ATRP method is promising as an excellent carrier for enzyme immobilization. This synthetic strategy can integrate versatile electrospun nanofibers with the densities and lengths of controllable polymer chains for more broadened applications.

## Figures and Tables

**Table 1:** Performance of the immobilized laccase using various immobilization methods and carriers.

Carriers	Method	Enzyme Loading	Reference
Amino-functionalized SBA-15 silica	Physical adsorption	57 mg/g	[43]
Halloysite nanotubes (HNTs) with Fe_3_O_4_ nanoparticles and chitosan	Physical adsorption	100.12 mg/g	[44]
Geopolymer	Physical adsorption	28.0 mg/g	[45]
PU/RC-poly (HEMA) nanofiber membrane	Ion coordination	84.21 mg/g	[33]
Monoaminoethyl-N-aminoethyl (MANAE–agarose)	Ionic adsorption	18 ± 0.5 mg/g	[46]
Concanavalin A-activated Fe_3_O_4_ nanoparticles	Ionic adsorption	29.4 mg/g	[47]
Sepharose-linked antibody	Covalent bonding	33 mg/g	[48]
Amidoxime polyacrylonitrile/montmorillonite (AOPAN/MMT) composite nanofibers	Covalent bonding	89.26 mg/g	[32]
Polystyrene-divinylbenzene-poly (glycidyl methacrylate) [PS-co-DVBg-P(CCMA)]-PGMA	Covalent bonding	47.8 mg/g	[49]
Polyurea microspheres	Covalent bonding	20.63 mg/g	[50]
polyamide 6/chitosan (PA6/CHIT) nanofibers modified by (i) bovine serum albumin (BSA) (ii) hexamethylenediamine (HMD)	Covalent bonding	(i) 64.1 ± 7.9 mg/g (ii) 72.9 ± 14.6 mg/g	[51]
Supermagnetized (Fe_3_O_4_) and chitosan (CS) functionalized halloysite nanotubes (HNTs) (Fe_3_O_4_-HNTs-CS)	Covalent bonding	90 mg/g	[52]
RC-poly (DMAEMA) nanofiber membrane	Physical adsorption	95.04 ± 4.35 mg/g	This work

## Data Availability

The data presented in this study are available on request from the corresponding author.
